# Maternal health infrastructure investments in Zambia: leveraging geospatial evidence to inform planning and prioritization

**DOI:** 10.1093/heapol/czag099

**Published:** 2026-08-10

**Authors:** Emmanuel Katyoka, Kutha Banda, Jason Wamulume, Patson Mwanza, Fredrick Mumba, Ibrahim Abdallah, Hilda Shakwelele, Olatubosun Akinola, Rabson Zimba

**Affiliations:** Clinton Health Access Initiative, Strengthening Accountability for Program Coordination and Acountability (SA&C) for the PeaCe health program, PO Box 51071, Ridgeway, Lusaka, Zambia; Clinton Health Access Initiative, Strengthening Accountability for Program Coordination and Acountability (SA&C) for the PeaCe health program, PO Box 51071, Ridgeway, Lusaka, Zambia; Ministry of Health, Department of Policy and Planning - Infrastructure Unit, Lusaka, Zambia; Ministry of Health, Department of Policy and Planning - Infrastructure Unit, Lusaka, Zambia; Clinton Health Access Initiative, Strengthening Accountability for Program Coordination and Acountability (SA&C) for the PeaCe health program, PO Box 51071, Ridgeway, Lusaka, Zambia; Clinton Health Access Initiative, Strengthening Accountability for Program Coordination and Acountability (SA&C) for the PeaCe health program, PO Box 51071, Ridgeway, Lusaka, Zambia; Clinton Health Access Initiative, Strengthening Accountability for Program Coordination and Acountability (SA&C) for the PeaCe health program, PO Box 51071, Ridgeway, Lusaka, Zambia; Clinton Health Access Initiative, Strengthening Accountability for Program Coordination and Acountability (SA&C) for the PeaCe health program, PO Box 51071, Ridgeway, Lusaka, Zambia; Clinton Health Access Initiative, Strengthening Accountability for Program Coordination and Acountability (SA&C) for the PeaCe health program, PO Box 51071, Ridgeway, Lusaka, Zambia

**Keywords:** geospatial analysis, maternal health, infrastructure investment, cost-efficiency, evidence-based planning, equity planning

## Abstract

In many low- and middle-income countries, inequities in maternal health outcomes are linked to disparities in spatial access to essential health services. In Zambia, significant portions of the rural population reside >5 km from the nearest health facility. This contributes to the ‘three delays’ of maternal mortality: delay to seek care, delay to reach a health facility, and delay to receive care from a skilled health provider. Consequently, at 187 per 100 000 live births, Zambia’s maternal mortality ratio is above the global target of <70. This paper describes the application of a geospatial prioritization approach used by the Ministry of Health to guide investments in new maternal health infrastructure (mothers’ waiting homes and maternity annexes) and upgrades to existing facilities. Using cross-sectional data from 692 public health facilities in Eastern and Southern Provinces, the approach integrates routine service and maternal mortality data from the national Health Management Information System , with facility infrastructure assessments and geospatial analysis. Facilities were screened using binary eligibility criteria based on recorded maternal deaths and then ranked using a weighted scoring model reflecting infrastructure readiness gaps for continuity of maternal care. Findings indicate widespread facility coverage for labor and delivery services, contrasted against inadequate capacity for the recommended 48-hr postnatal observation care. Maternal deaths were concentrated in 28% of facilities in Eastern and 19% in Southern Province. Applying the prioritization framework reduced the potential scope of infrastructure investment from 606 facilities to 27 high-priority sites, lowering projected investment costs from US$41.8 million to ∼US$2 million. The approach demonstrates how targeted infrastructure investments, informed by geospatial and routine health system data, can improve equity and continuity of care and optimize impact from limited resources. This applied framework offers a practical and scalable model for evidence-informed infrastructure planning in resource-constrained settings.

Key messages

- In resource-constrained health systems such as Zambia and most of sub-Saharan Africa, decision models based on geospatial prioritization have the potential to offer an evidence-based way to identify and rank locations where investments in maternal health infrastructure can improve equity of access, improve outcomes, and ensure every dollar of investment is directed where it can have the greatest impact.
- Integrating geospatial analysis, routine service data, and a robust prioritization model provides a clear route for planners to improve spatial access to maternal and other health services by directing infrastructure investments to the most underserved and high-risk areas, thereby advancing health equity in both service availability and outcomes in a systematic, transparent, and resource-based manner.
- Despite facilities having a high coverage of antenatal, labor, and delivery services, in the Eastern and Southern Provinces, only 31% of these facilities had adequate capacity for the critical required 48 hr of postnatal care. Overlay of GIS coordinates of facility locations and maternal outcomes visually highlights how infrastructure availability deficits compromise care continuity and increase the risk of poor maternal and neonatal outcomes. The successful implementation in Zambia demonstrates operational feasibility. However, scaling nationally and to other contexts requires balancing efficiency gains with equity safeguards.

## Introduction

Globally, an estimated 810 women die daily from preventable causes related to pregnancy and childbirth. Overall, 95% of these deaths occur in low- and middle-income countries (World Health Organization 2019)

In Zambia, the mortality ratio is 187 deaths per 100 000 live births (The DHS Program – Zambia 2024), above the global target of <70 deaths per 100 000. Most of these are preventable and attributable to the ‘three delays’ of maternal mortality postulated by Thaddeus and Maine (1994): delays to seek care; delays to reach a health facility; and delays to access services from qualified health workers (Thaddeus and Maine 1994), underscoring the importance of timely access to skilled care, which is often constrained by physical distance, poor referral systems, and inadequate facility readiness (Thaddeus and Maine 1994).

Spatial barriers, particularly travel time to delivery-ready facilities, have been linked to poor maternal health outcomes (Lohela et al. 2012). In Zambia, spatial access to health facilities is uneven, with near-universal coverage within 5 km in urban areas and substantially lower coverage in rural areas. Even where facilities are spatially accessible, including most urban areas, insufficient maternity bed capacity and limited spaces for postnatal observation undermine continuity of care for pregnant women, most of whom travel long distances to reach delivery-ready facilities.

The Ministry of Health (MOH), in collaboration with the Clinton Health Access Initiative (CHAI), implemented a geospatial prioritization approach aimed at identifying optimal locations where targeted investments in the construction of new mothers’ waiting homes (to minimize travel times and increase monitoring of pregnant women for complications before delivery) and maternity wards (to provide bed spaces for labor, delivery services, and the critical 48 hr of postnatal care) yield the greatest maternal health impact under constrained resource conditions.

### Implementation

The approach employed a mixed-methods design, incorporating a cross-sectional assessment of 692 health facilities across 27 districts in the Eastern and Southern Provinces of Zambia, spanning both rural and urban settings. Data was collected on service delivery capacity, including delivery beds, postnatal observation space, and the presence of maternity wards or maternal waiting shelters. Mortality data were extracted from the Health Management Information System (HMIS). Geospatial coordinates were collected using GPS-enabled devices and validated against the District Health Information Software 2 (DHIS2) facility registry.

Geospatial data identified facilities that recorded a maternal death during the assessment period. This served as a first-level eligibility filter, recognizing maternal deaths as sentinel indicators of service delivery failures rather than as epidemiological risk estimates.

Facilities meeting the following criteria were assessed as additional factors: (i) presence of a maternity ward or mothers’ shelter, (ii) adequacy of delivery bed spaces, and (iii) availability of bed space for the critical 48-hr period of postnatal care.

Availability of essential maternal health commodities and skilled birth attendants was held constant, as these inputs were outside the scope of the infrastructure intervention and were mandated to be provided through existing MOH systems.

A descriptive geospatial overlay of maternal death occurrence and infrastructure deficits was used to visually identify priority zones where high mortality burden coincided with significant service readiness gaps. No formal spatial statistical or hotspot detection techniques were applied; the geospatial analysis was designed as a visualization tool to support planning discussions. A binary decision rule and a weighted scoring model were developed to guide site selection and sequencing of maternal health infrastructure investments under constrained resource conditions. The decision rule identified locations for prioritization, while the weighted scoring model ranked eligible facilities based on infrastructure readiness gaps.

### Weighted scoring model

For facilities meeting the eligibility criteria, an overall priority score was calculated using the following formula:


$$Score=X_{0}D+X_{1}I+X_{3}B+C+S$$

Where D = 1 if a maternal death was recorded, 0 otherwise; I = 1 if maternal inpatient services are available, 0 otherwise; B = 1 if bed space is adequate to retain women for up to 48 hr post-delivery, 0 otherwise; C = essential commoditilability was held constant; S = 1 if there is adequate space to accommodate women postnatally, 0 otherwise; and X_0_, X_1_, X_3_ represent proportional weights assigned to maternal deaths, inpatient service availability, and bed capacity, respectively. Weights were derived based on each indicator’s relative contribution to the overall infrastructure and service readiness gaps observed across the two Provinces, rather than through statistical optimization.

The model was used to rank and sequence facilities that met the minimum eligibility criteria. It was not intended to predict maternal outcomes or generate continuous risk estimates but to support transparent prioritization under budget constraints.

### Binary prioritization rule

A binary prioritization rule classifies facilities as either prioritized or not for infrastructure investment.

The rule was defined as:


$$\begin{array}{rl} & P=\{\begin{array}{rl} 1 & \;\mathrm{if}\;D=1\;\mathrm{and}\;[I=1\;\mathrm{and}\;B=0)\;\mathrm{or}\;(I=0\;\mathrm{and}\;(C=0\;\mathrm{or}\;S=0))\\ & \;\mathrm{or}\;(I=1\;\mathrm{and}\;B=1)]\\ 0 & \;\mathrm{otherwise} \end{array} \end{array}$$

where P = 1: facility is prioritized for infrastructure investment, and where P = 0: facility is not prioritized.

This rule ensured that facilities that recorded maternal deaths were prioritized regardless of whether infrastructure gaps related to inpatient service availability, postnatal bed capacity, or accommodation space, reflecting the program’s emphasis on preventing repeat adverse outcomes.

### Cost estimation

The final step involved estimating the cost of constructing new mothers’ waiting homes, making improvements in water and electricity at existing facilities, and constructing new maternity wards (annexes) to increase bed spaces for delivery and postnatal care.

### Adaptation, implementation scale, and stakeholder engagement

The MOH implemented the model in two Provinces with technical support from the CHAI. To ensure ownership, the data were validated, and the prioritization outputs were contextualized by national, provincial, and district-level health officers.

This iterative engagement process ensured that analytical outputs informed, rather than replaced, routine planning and budgeting decisions.

## Results

The adopted approach innovates away from traditional planning methods that rely on composite scores or demographic thresholds. It employs a geospatial triage mechanism to identify facilities requiring urgent investment and highlights maternal deaths as critical indicators of health system failure. Additionally, the model integrates equity and epidemiological urgency within a weighted prioritization framework, creating a replicable approach for proactive infrastructure investment prioritizing equity and access for vulnerable populations.

### Findings of the service availability and infrastructure readiness assessments

Assessment covered 692 facilities, with a combined catchment population of 4 916 626. i.e. 327 facilities with a catchment population of 2 475 819 in the Eastern province and 365 facilities with a catchment population of 2 440 807 in the Southern province. Findings revealed high service availability for antenatal, labor, and delivery services, against a backdrop of critical infrastructure shortfalls and heavy clustering of maternal deaths in ∼28% of facilities in Eastern and 19% in Southern Provinces.

#### Service availability and readiness

Antenatal care was provided at 98% of facilities, while postnatal services were offered at 99% in Eastern and Southern Provinces. Labor and delivery services were available in 91% of facilities in Eastern and 85% in Southern provinces (Fig. 1)

**Figure 1:**
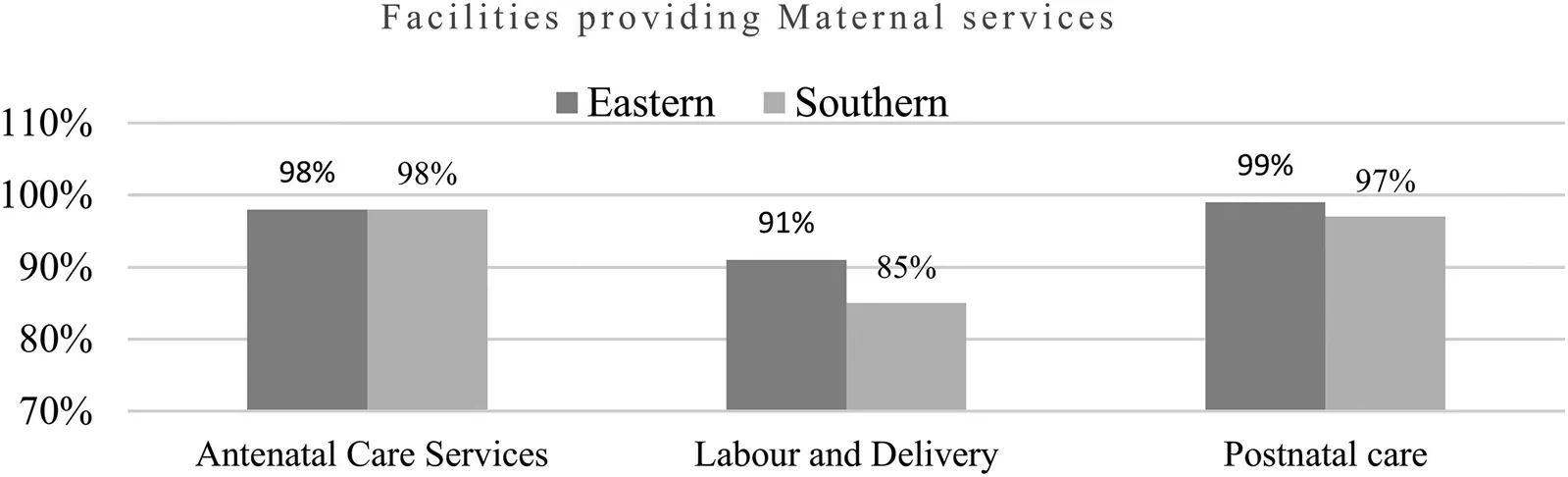
Facilities providing maternal services.

#### Infrastructure readiness

Infrastructure service readiness was assessed from: (i) bed spaces availability to retain women for the recommended 48 hr of critical postnatal care and (ii) availability of a dedicated maternity annex (Fig. 2). Overall, 72% of facilities in Eastern and 65% in Southern Provinces lacked adequate room for 48 hr of postnatal care.

**Figure 2:**
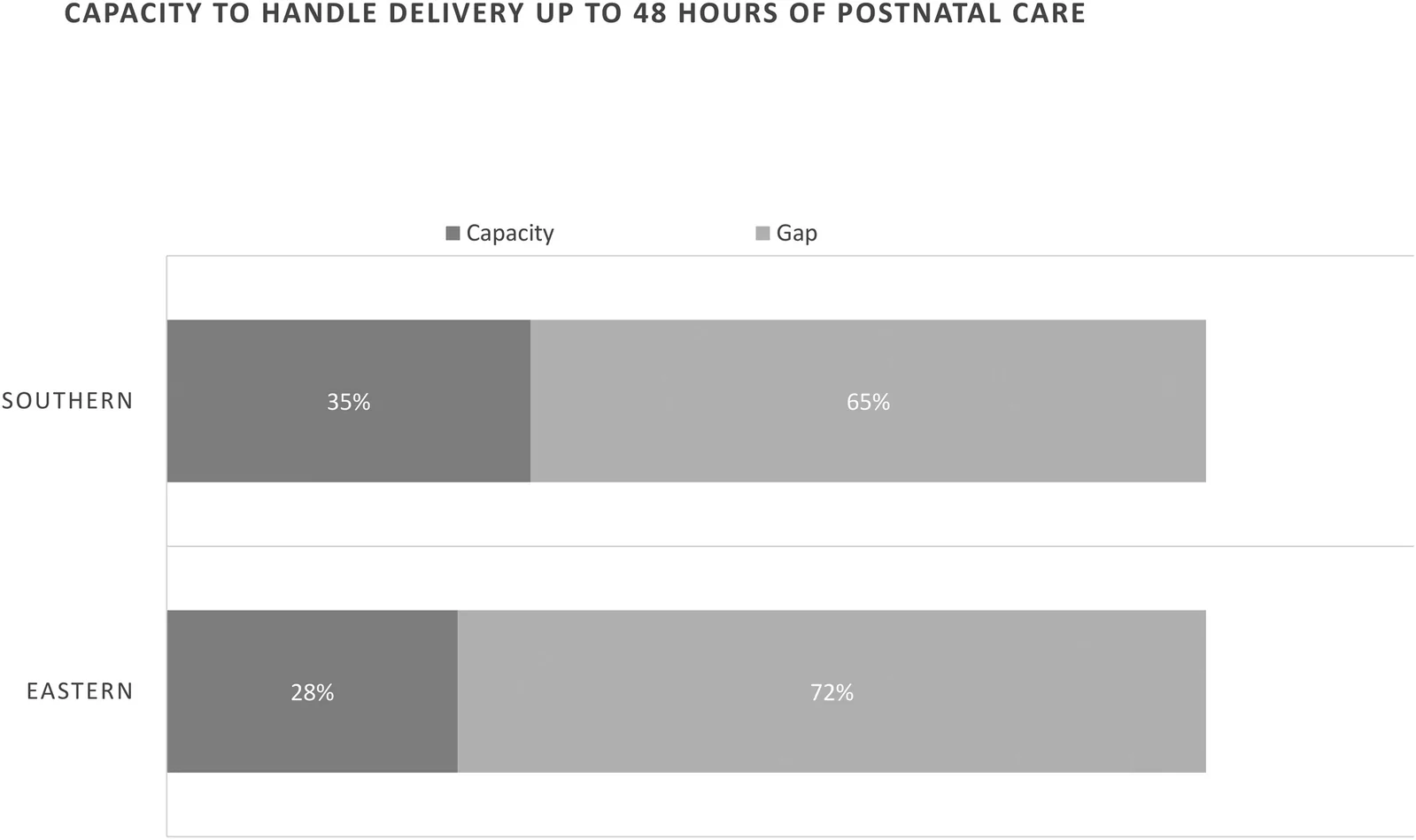
Capacity to handle delivery of up to 48 hr of postnatal care.

### Availability of a dedicated delivery room or maternity annex

In the Eastern province 32% (*n* = 117/365) of facilities had a maternity ward, while in the

Southern province 29% (*n* = 94/327) of facilities had a maternity ward.

### Maternal outcome

HMIS data revealed significant clustering of maternal deaths in both provinces. Maternal deaths were clustered in ∼28% of facilities in the Eastern Province and 19% in the Southern Province

### Geospatial overlay

Geospatial overlay of coordinates for sites where maternal deaths occurred over sites with infrastructure challenges aided in the identification of maternal death hotspots at district and facility levels.

### Spatial overlay—Eastern Province

Figure 3 shows the spatial overlay for the Eastern Province, showing 8 out of 14 districts accounting for all maternal deaths in the province. These deaths were traced to 28% (91/327) of facilities in the province. Spatial overlay for maternal and neonatal death burden against the backdrop of infrastructure deficits is also shown in Fig. 3.

**Figure 3:**
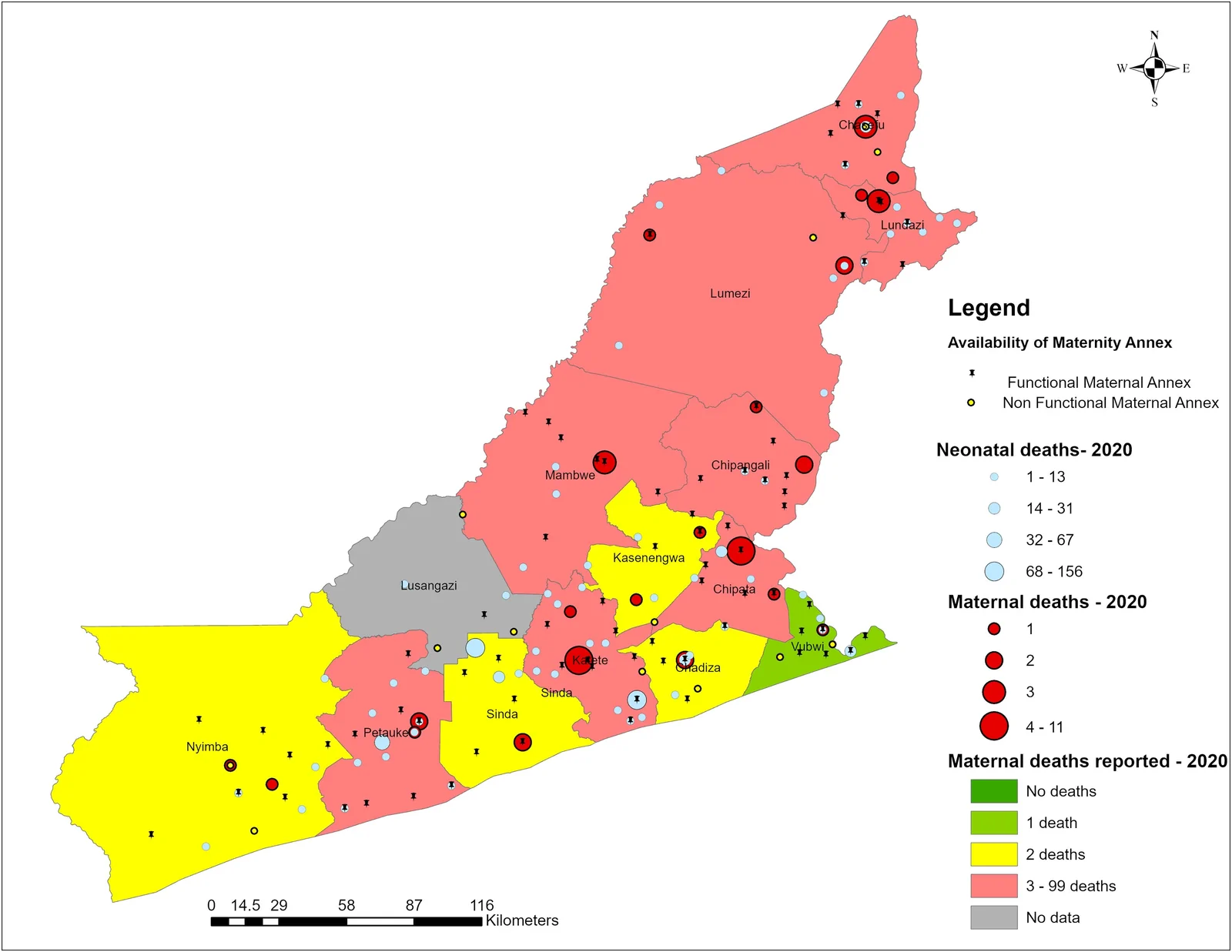
Spatial overlay, maternal, and neonatal death burden against the backdrop of infrastructure deficits in Eastern Province.

### Spatial overlay—–Southern Province

Figure 4 showing the spatial overlay for the Southern province illustrates the eight 8 out of 14 districts accounting for all maternal deaths. These deaths were traced to 64 facilities, representing 19% (64/327). Triangulation of facility infrastructure gap analysis data showed that of the 64 facilities that presented a maternal death, 14 sites, representing 22% of facilities, also lacked a dedicated maternity annex

**Figure 4:**
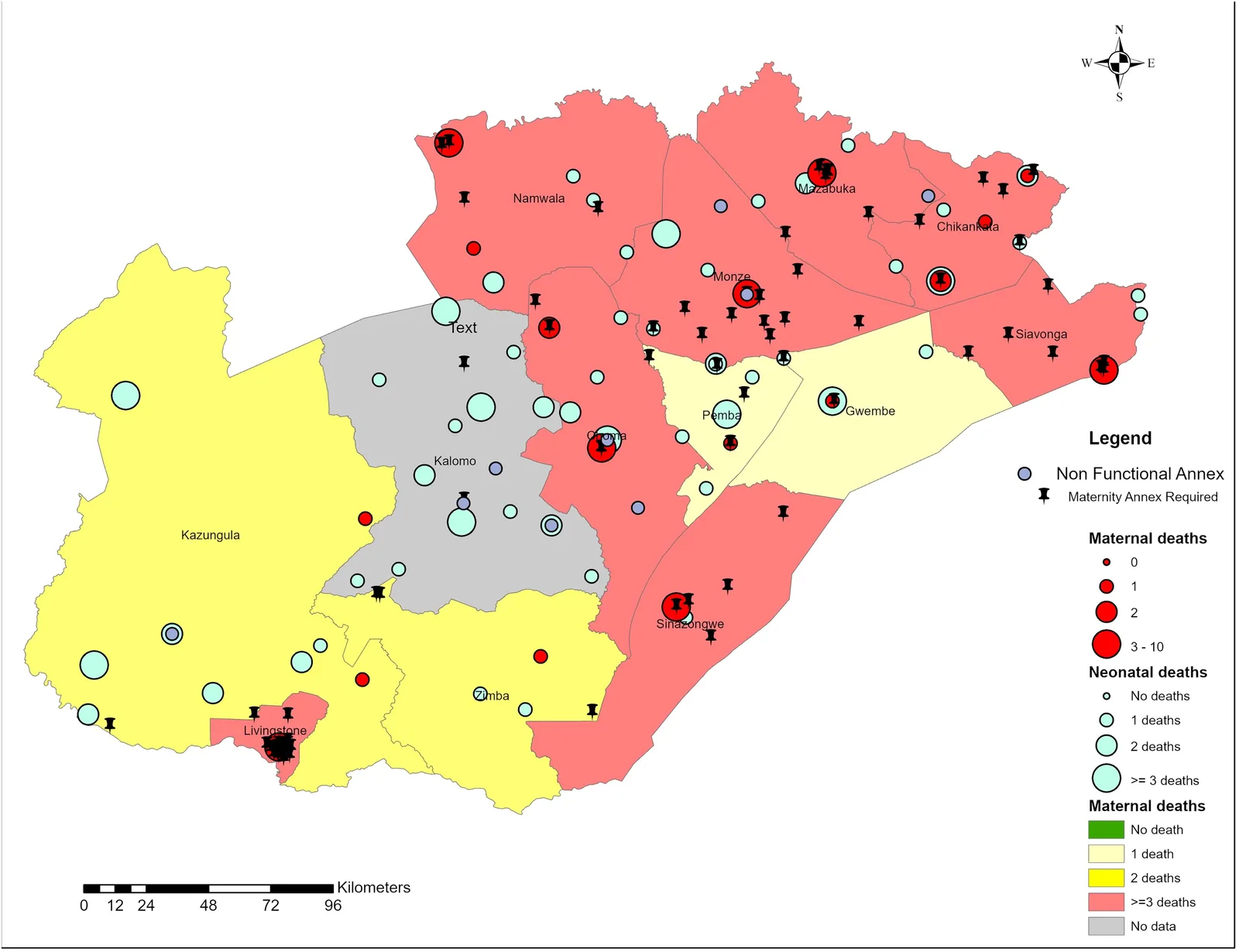
Spatial overlay, maternal, and neonatal deaths against the backdrop of infrastructure deficits in Southern Province.

### Facility prioritization for infrastructure investment

Initial criteria identified 606 (out of 692) facilities as providing labor and delivery services; 155 (25%) were identified as high maternal death burden facilities. After accounting for physical space availability and mortality burden, 27 (5%) facilities were identified as having both maternal burden and lacking dedicated space to support labor and delivery services and the required 48 hr of postnatal care. These were ranked high-priority sites for infrastructure investment, i.e. construction of mothers waiting homes and maternity annexes and rehabilitation of existing facilities (Fig. 5).

**Figure 5:**
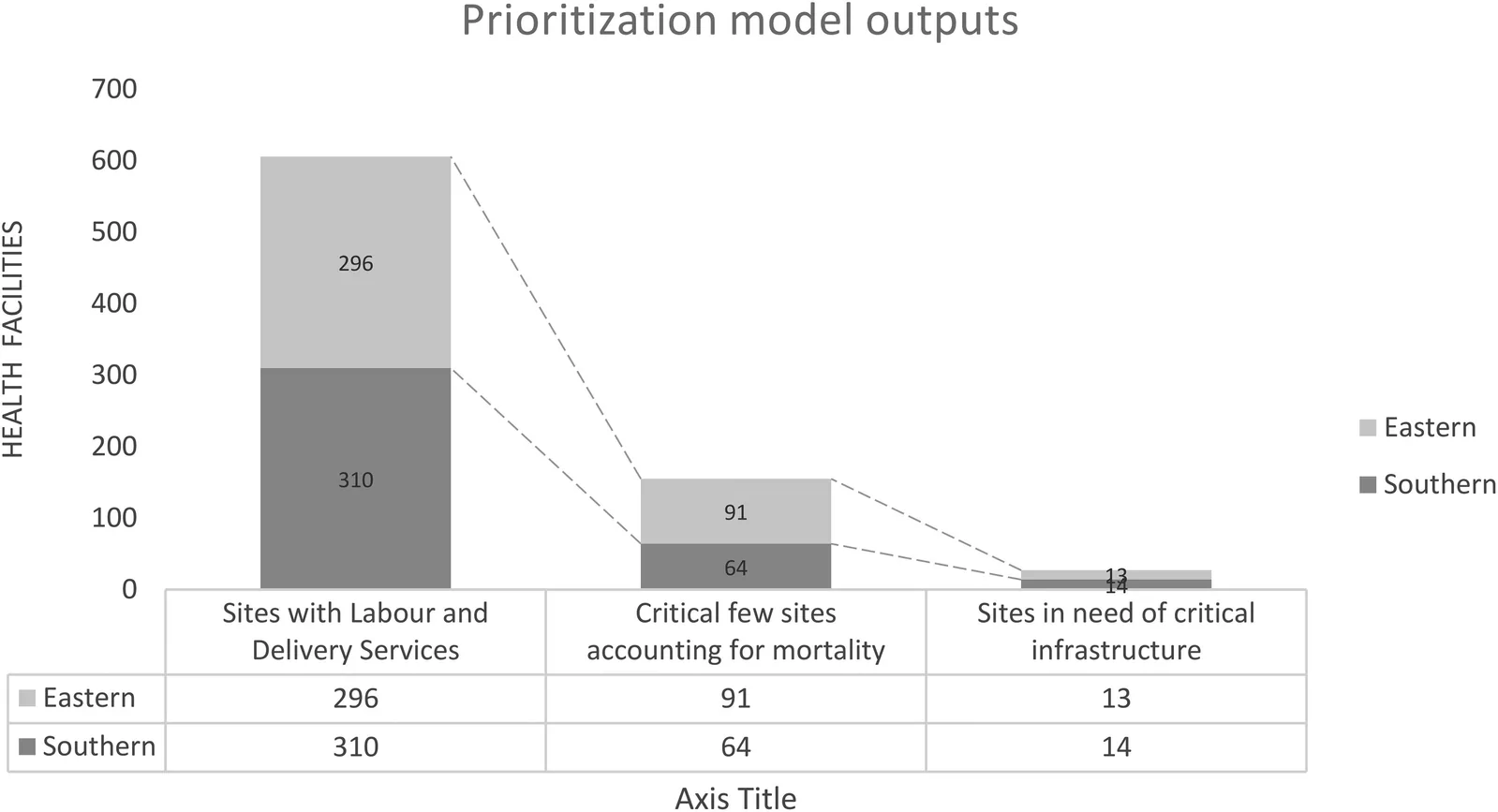
Prioritization model outputs.

#### Infrastructure investment cost estimation

Engineering cost assessments projected a total investment of $41.8 million to construct new mothers’ waiting homes and maternity annexes and upgrade all 155 sites, and after prioritisation, this reduced to $2 million for the 27 high-priority sites

### Achievements/challenges

This work innovates by integrating a weighted scoring model with a binary prioritization rule layered onto spatial data. This addresses a common challenge in low- and middle-income countries’ health systems: how to objectively rank facilities for investment under constrained resource settings. Using this approach reduced the initial pool of candidate facilities from 606 to 27, out of a total of 697 facilities, without compromising equity or geographic coverage. The approach also lowered projected investment costs from $41.8 million to $2 million, significantly enhancing cost efficiency.

### Challenges

#### Tradeoffs between equity and efficiency

The prioritization model excluded many underserved facilities that did not meet mortality or readiness thresholds, raising the policy dilemmas about balancing efficiency with spatial and equity concerns.

#### Data limitations

Infrastructure indicators were limited to availability of dedicated spaces and bed space capacity. In practice, other factors, such as the availability of skilled health workers, affect outcomes.

### Enablers

The availability of routine data systems HMIS and GIS data enabled triangulation of maternal death data, service availability, and readiness.

#### Policy and planning implications

Scalability: the methodology is scalable to other provinces and adaptable to different health service areas (e.g. child health, emergency care). Institutionalization: embedding spatial analytics into the MOH’s planning cycle can institutionalize evidence-based decision-making. Equity focus: geospatial approaches help identify underserved areas, supporting equitable health service distribution.

### Constraints/limitations

The descriptive spatial overlay approach lacks the rigor of formal hotspot statistical techniques. The analysis was limited to Eastern and Southern Provinces, limiting generalizability to national contexts. Key determinants of maternal outcomes (e.g. supply chain, staffing) were excluded, potentially oversimplifying facility readiness. While the approach points to high-impact investment sites, in practice, priorities for infrastructure are modulated by other considerations, including political considerations.

### Future directions

The implications of travel time should be incorporated to understand access barriers better; real-time data systems should be used to support dynamic resource planning; and the approach should be expanded to a national scale to inform broader infrastructure policy.

## Conclusion

Geospatial analytics, when integrated with prioritization models and decision rules, can transform how government planners use routine data from HMIS to identify, target, and sequence health infrastructure investments using an equity lens.

By identifying where needed investments potentially yielded the highest service and outcome impact, investment outlays were reduced by $39 million, without sacrificing equity, demonstrating that: (i) routine service and outcome data systems used may not be perfect, however, they enhance intervention targeting and aid precision planning; (ii) integrating health, engineering, and spatial data ensures that prioritization reflects need and feasibility; and (iii) where equity concerns are strong, efficiency-focused planning must be accompanied by clear communication on why some facilities are prioritized over others.

## Data Availability

The data underlying this article will be shared on reasonable request to the corresponding author, and the Clinton Health Access Initiative.

## Reflexivity statement

This paper draws on the collective experience of the authors as technical and implementation partners within the People at the Centre (PeaCe) Health Program, a health systems strengthening initiative led by the Zambian Ministry of Health in the Eastern and Southern Provinces, with technical support from the Clinton Health Access Initiative (CHAI), and funded by the Government of Sweden. This paper’s research and practice-based insights were generated through direct engagement in program design, implementation, and monitoring in a resource-constrained setting. The authors represent a multidisciplinary team working across geospatial analysis, infrastructure planning, health systems strengthening, and program coordination. Geospatial modelling and spatial analytics were led by R.Z. and F.M.; E.K., J.W., and P.M. contributed infrastructure assessment and prioritization; H.S., O.A., and I.A. guided health systems strategy and intervention planning; while E.K., R.Z., and K.B. supported data analysis and synthesis of findings. The authors bring varied institutional and professional backgrounds to this work. E.K. is a process engineer and infrastructure associate working to enhance program coordination and accountability under PeaCe. R.Z. operates at a mid-senior technical level. O.A., I.A., and H.S. are senior public health leaders with policy and system design expertise. P.M. and J.W. are career infrastructure professionals with extensive tenure in the Ministry of Health. K.B. is a public health and knowledge management specialist contributing to learning and dissemination efforts. F.M. is a monitoring and evaluation specialist.
